# Echo response and clinical outcome in CRT patients

**DOI:** 10.1007/s12471-015-0767-5

**Published:** 2015-12-07

**Authors:** J. van ’t Sant, T.P. Mast, M.M. Bos, I.A. ter Horst, W.M. van Everdingen, M. Meine, M.J. Cramer

**Affiliations:** Department of Cardiology, University Medical Center Utrecht, Heidelberglaan 100, 3584 CX Utrecht, The Netherlands

**Keywords:** Cardiac resynchronisation therapy, Surrogate markers

## Abstract

**Background:**

Change in left ventricular end-systolic volume (∆LVESV) is the most frequently used surrogate marker in measuring response to cardiac resynchronisation therapy (CRT). We investigated whether ∆LVESV is the best measure to discriminate between a favourable and unfavourable outcome and whether this is equally applicable to non-ischaemic and ischaemic cardiomyopathy.

**Methods:**

205 CRT patients (age 65 ± 12 years, 69 % men) were included. At baseline and 6 months echocardiographic studies, exercise testing and laboratory measurements were performed. CRT response was assessed by: ∆LVESV, ∆LV ejection fraction (LVEF), ∆ interventricular mechanical delay, ∆VO_2_ peak, ∆VE/VCO_2_, ∆BNP, ∆creatinine, ∆NYHA, and ∆QRS. These were correlated to the occurrence of major adverse cardiac events (MACE) between 6 and 24 months.

**Results:**

MACE occurred in 19 % of the patients (non-ischaemic: 13 %, ischaemic: 24 %). ∆LVESV remained the only surrogate marker for CRT response for the total population and patients with non-ischaemic cardiomyopathy, showing areas under the curve (AUC) of 0.69 and 0.850, respectively. For ischaemic cardiomyopathy, ∆BNP was the best surrogate marker showing an AUC of 0.66.

**Conclusion:**

∆LVESV is an excellent surrogate marker measuring CRT response concerning long-term outcome for non-ischaemic cardiomyopathy. ∆LVESV is not suitable for ischaemic cardiomyopathy in which measuring CRT response remains difficult.

## Background

Cardiac resynchronisation therapy (CRT) reduces major adverse cardiac events (MACE), and improves exercise capacity and functional class, in drug refractory heart failure patients (NYHA ≥ II) with ventricular conduction delay [1–3]. However, the degree of CRT response varies widely and ranges from MACE rates similar to heart failure patients *without* CRT to rates comparable with a matched sample from the general population [4, 5]. In daily clinical practice, degree of CRT response is assessed 6 months after CRT implantation using echocardiographic change (∆) of left ventricular end-systolic volume (LVESV) [6]. However, whether ∆LVESV is the most appropriate surrogate marker has not been extensively investigated. Furthermore, due to differences in underlying disease, response measures might not be equally appropriate for patients with non-ischaemic and ischaemic cardiomyopathy.

Two basic criteria need to be fulfilled for a variable to be a suitable surrogate marker: (1) A relation has to be demonstrated between the *change* of the surrogate marker over time (e.g. LVESV decrease) and the true endpoint (MACE), and (2) a pathophysiological foundation must be known to consider the surrogate marker as a main determinant of the true endpoint [7]. LVESV declines after CRT due to reverse remodelling and has been demonstrated to correlate with a favourable prognosis [8, 9]. Besides change in LV volume, changes in parameters indicating LV systolic function, mechanical dyssynchrony, cardiopulmonary condition and biomarkers of overall cardiovascular function are potential surrogate markers for CRT response [10–17].

In this study we investigated (1) several surrogate markers for CRT response, and (2) whether these surrogate markers were equally applicable for non-ischaemic and ischaemic cardiomyopathy.

## Methods

### Study design and cohort

This was a retrospective study in which we included 205 consecutive patients who received a CRT device (200 received a CRT defibrillator and four a CRT pacemaker) in the University Medical Center Utrecht (UMCU) between August 2005 and August 2011, with prospectively planned echocardiographic studies, cardiopulmonary exercise testing and laboratory evaluation available before implantation. It was recommended not to change device settings during the first 6 months. Configurations were only adjusted by exception, during regular check-up at 2 months. Thereafter, until the six-month point of assessment, pacing configurations remained unchanged.

Six months after device implantation, evaluation of the echocardiographic studies, cardiopulmonary exercise testing and laboratory tests was repeated. To determine the best surrogate marker for CRT response, the correlation between all potential surrogate CRT response measurements and MACE occurring between 6 and 24 months was assessed. We did not include MACE occurring before our six-month evaluation point, as therapy effect was quantified at 6 months in order to gather prognostic information for the period afterwards, not the preceding period. In addition, the effect of CRT might not yet be optimal in the preceding period, which was another reason why we chose not to include MACE occurring in the first 6 months to correlate to surrogate response measurements. The study was conducted in accordance with the Declaration of Helsinki.

Response to CRT was assessed 6 months after device implantation by (1) ∆LVESV (%), (2) ∆left ventricular ejection fraction (LVEF; absolute %), (3) ∆interventricular mechanical delay (IVMD; ms), (4) ∆VO_2_ peak (ml/kg/min), (5) ∆ percentage of predicted VO_2_ peak (absolute %), (6) ∆VE/VCO_2_ slope, (7) ∆brain natriuretic peptide (BNP; pmol/L), (8) ∆creatinine (µmol/L), ∆NYHA, and ∆QRS duration.

### Echocardiography

Data were acquired using Philips IE 33 (Philips Medical Systems, Andover, Massachusetts, USA) or Vivid 7 (General Electric, Milwaukee, USA) ultrasound machines. Echocardiographic parameters were assessed using Xcelera software (R3.3L1). Volumes and ejection fraction were assessed by Simpsons’ biplane method in accordance with the guidelines of the American Society of Echocardiography (ASE) and European Association of Echocardiography (EAE) [10].

Doppler flows over the pulmonary and aortic valve were recorded and time from Q to onset of flow was assessed for both valves. IVMD was defined as the time span between opening of the aortic valve and the pulmonary valve [6]. Measurements were performed on three separate beats, or five beats in case of irregular rhythms.

### Cardiopulmonary exercise testing

Cardiopulmonary exercise testing on the bicycle ergometer started with unloaded cycling for 2 min after which a protocol of stepwise incremental exercise was applied, starting at 25 W with increments of 5, 10 or 15 W every minute depending on the estimated maximum workload. Rotation speed was kept around 60 per minute. Prior to testing, gas and flow sensors were calibrated utilising gases with established concentrations of O_2_ and CO_2_.

Ventilation (VE) (L/min), peak oxygen consumption (VO_2_) (mL/kg/min), and carbon dioxide production (VCO_2_) (L/min) were measured for each patient throughout the exercise on a breath-to-breath basis. VO_2_ peak and VE/VCO_2_ slope were assessed. Data for the VE/VCO_2_ slope were collected throughout the exercise, with exclusion of the unloaded cycling and recovery period.

VO_2_ peak is the level of O_2_ consumption during maximum effort and averaged over a 30-second period [11]. The VE/VCO_2_ slope provides a measure of CO_2_ exchange efficiency by assessing required ventilation for CO_2_ elimination [11].

### Long-term follow-up and assessment of major adverse cardiac events

MACE was defined as: hospital admission for heart failure, sustained ventricular tachycardia (VT) (VT lasting > 30 s or a VT beneath the monitoring zone leading to hospitalisation), ventricular fibrillation (VF), receiving a left ventricular assist device (LVAD), undergoing heart transplantation, death due to heart failure and appropriate implantable cardioverter defibrillator (ICD) shocks (due to VT or VF). Multicentre studies proved a CRT defibrillator was beneficial over a CRT pacemaker device concerning long-term survival, indicating that the shock function of the defibrillator prevents MACE in a heart failure population and therefore an appropriate shock was considered as an adverse cardiac event [12].

### Statistical analyses

Statistical analysis was performed using SPSS version 20.0 (SPSS Inc., Chicago, Illinois). Continuous variables are presented as mean with standard deviation (SD) when normally distributed (mean ± SD) and as median with interquartile range (IQR) in case of non-normal distribution (median (IQR)). Distributions were checked using Q-Q plots. Categorical variables are presented as numbers and percentages.

Surrogate markers of CRT response were assessed for the total population, and the ischaemic and non-ischaemic subpopulations. Data were compared with a T-test or Mann-Whitney U test in case of non-normal distribution. A *p*-value of < 0.05 was considered statistically significant. Surrogate markers demonstrating a significant difference between patients with and without MACE were subsequently tested using univariable and multivariable Cox backward logistic regression analyses. Areas under the receiver-operating curve (AUC) were assessed with respect to the occurrence of MACE. Subsequently, Cox regression multivariable analyses were performed to evaluate the association between significant surrogate markers and time to first MACE. These analyses were performed for the total population, ischaemic and non-ischaemic subpopulations. Multicollinearity amongst variables was checked using variance inflation factors, and a value of > 5 was considered evidence of multicollinearity. Furthermore, the relation between surrogate markers of CRT response and MACE was calculated by the Kaplan-Meier method and compared by means of the log-rank test. A two-tailed probability value of *p* < 0.05 was considered statistically significant.

## Results

### Baseline results

Baseline characteristics are listed in Table 1. Mean age 65 ± 12 years, 69 % (*n* = 142) male, 52 % (*n* = 106) with ischaemic cardiomyopathy. At baseline, LVESV was 180 (87) mL, LVEF 22 ± 7 %, and IVMD 46 ± 28 ms. Baseline VO_2_ peak was 14 ± 4 ml/kg/min, predicted VO_2_ peak 61 ± 19 % and VE/VCO_2_ slope 38 (15). Baseline BNP levels were 112 (172) pmol/L and creatinine levels were 112 (52) µmol/L.

**Table 1:** Baseline and six-month follow-up parameters

	Baseline (*n* = 205)	Six-month FU (*n* = 197)	*P*-value
**Clinical**			
Age, mean ± SD (years)	64.8 ± 12.4	–	
Male gender (%)	142 (69)	–	
Ischaemic cardiomyopathy (%)	106 (52 %)	–	
NYHA I (%)	1 (0.5)	16 (8.3)	0.001
NYHA II (%)	26 (12.7)	104 (53.6)	0.171
NYHA III (%)	167 (81.5)	71 (36.6)	0.062
NYHA IV (%)	11 (5.3)	3 (1.5)	< 0.001
**ECG**			
QRS duration, mean ± SD, ms	166 ± 24	153 ± 24	< 0.001
Left bundle branch block (%)	116 (57)	–	
Interventricular conduction delay (%)	63 (31)	–	
Right bundle branch block (%)	1 (0.5)	–	
QRS < 120 ms (%)	1 (0.5)	–	
Right ventricular pacing (%)	23 (11)	–	
**Echocardiography**			
LVESV, median (IQR), ml	180 (87)	133 (95)	< 0.001
LVEDV, median (IQR), ml	230 (92)	182 (115)	< 0.001
LVEF, mean ± SD (%)	21.6 ± 6.8	28.6 ± 10.4	< 0.001
IVMD, mean ± SD (ms)	46 ± 28	15 ± 28	< 0.001
**Exercise test**			
Peak VO_2_, mean ± SD (ml/kg/min)	14.0 ± 4.2	15.4 ± 4.8	< 0.001
Percentage of predicted peak VO_2_, mean ± SD (%)	61.1 ± 18.8	67.4 ± 23.2	< 0.001
VE/VCO_2_, median (IQR)	38 (15)	34 (11)	< 0.001
**Laboratory tests**			
BNP, median (IQR), pmol/L	112 (172)	71 (107)	< 0.001
Creatinine, median (IQR), µmol/L	112 (52)	115 (53)	0.002
**Medication**			
Beta-blocker (%)	157 (78)	–	
ACE-inhibitor (%)	152 (76)	–	
Diuretics (%)	180 (90)	–	

*BNP* brain natriuretic peptide, *FU* follow-up, *IVMD* interventricular mechanical delay, *LVEF* left ventricular ejection fraction, *LVEDV* left ventricular end-diastolic volume, *LVESV* left ventricular end-systolic volume, *NYHA* New York Heart Association, *VO*_*2*_ oxygen consumption, *VE/VCO*_*2*_ CO_2_ exchange efficiency.

### Six-month results

Six-month follow-up values are listed in Table 1. At six-month follow-up LVESV was 133 (95) ml, LVEF 29 ± 10 %, and IVMD was 15 ± 28 ms. VO_2_ peak was 15 ± 5 ml/kg/min, predicted VO_2_ peak 67 ± 23 % and VE/VCO_2_ slope 34 (11). BNP levels were 71 (107) pmol/L and creatinine levels were 115 (53) µmol/L.

### Long-term follow-up and major adverse cardiac events

**Figure 1:**
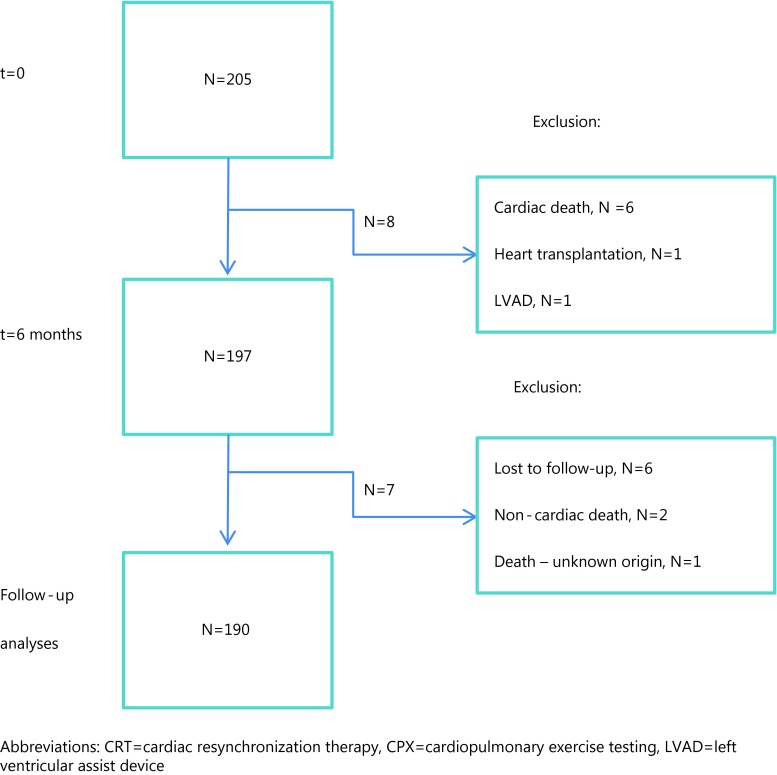
Flow chart of inclusion. CRT therapy between 2005–2011 and prospectively planned baseline and 6 months CPX and echocardiography. *CRT* cardiac resynchronization therapy, *CPX* cardiopulmonary exercise testing, *LVAD*  left ventricular assist device

Six patients died before six-month follow-up, one patient had undergone a heart transplant and one received an LV assist device; these patients were not included for further analyses. Of the remaining 197 patients, four patients were lost to follow-up, two died of a non-cardiac cause and one died of an unknown cause (Fig. 1). Between 6 and 24 months 19 % (36/190) experienced at least one MACE. Twenty patients had a hospital admission due to heart failure, nine had ventricular arrhythmias, and seven died due to heart failure. Of the ischaemic patients 24 % (*n* = 24) experienced a MACE versus 13 % (*n* = 12) of non-ischaemic patients (*p* = 0.061).

### Assessment of CRT response

**Figure 2:**
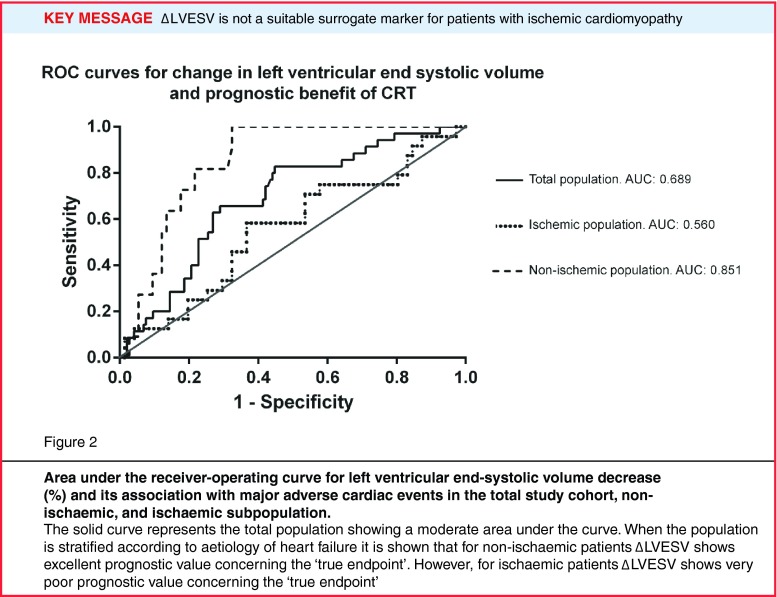
**Area under the receiver-operating curve for left ventricular end-systolic volume decrease (%) and its association with major adverse cardiac events in the total study cohort, non-ischaemic, and ischaemic subpopulation**. The solid curve represents the total population showing a moderate area under the curve. When the population is stratified according to aetiology of heart failure it is shown that for non-ischaemic patients ∆LVESV shows excellent prognostic value concerning the ‘true endpoint’. However, for ischaemic patients ∆LVESV shows very poor prognostic value concerning the ‘true endpoint’

∆LVESV, ∆LVEF, and ∆BNP differed significantly between patients with and without MACE in the total population (Table 2). After multivariable analyses, LVESV remained the only surrogate marker for CRT response significantly correlated to the occurrence of MACE. Discriminative performance of this surrogate marker was moderate: AUC = 0.69 (Table 3 and Fig. 2).

**Table 2:** Comparison of changes in echocardiographic, cardiopulmonary, and laboratory parameters between patients with and without a MACE

Potential surrogate endpoints	MACE (*n* = 36)	Non-MACE (*n* = 154)	*P*-value
**∆ LVESV (%)**	− 9.5 ± 18.7	− 23.5 ± 23.0	< 0.001
**∆ LVEF (absolute %)**	3.5 ± 6.2	8.2 ± 9.0	< 0.001
**∆ IVMD (ms)**	− 29 ± 35	− 34 ± 32	0.422
**∆ QRS duration (ms)**	4.0 ± 28.4	16.4 ± 26.3	0.022
**∆ Peak VO**_**2**_ **(ml/kg/min)**	0.8 ± 3.0	1.1 ± 3.1	0.684
**∆ Predicted peak VO**_**2**_ **(absolute %)**	5.7 ± 14.7	6.0 ± 14.8	0.930
**∆ VE/VCO**_**2**_ **slope**	− 2 (17)	− 4 (9)	0.674
**∆ BNP (pmol/L)**	14.5 (125)	− 21.5 (106)	0.019
**∆ Creatinine (µmol/L)**	0.5 (23)	− 4.8 (22)	0.375
**NYHA improvement (%)**	12 (38)	94 (63 %)	0.009

*∆* indicates a change, *BNP* brain natriuretic peptide, *ICM* ischaemic cardiomyopathy, *IVMD* interventricular mechanical delay, *LVEF* left ventricular ejection fraction, *LVESV* left ventricular end-systolic volume, *MACE* major adverse cardiac events, *NYHA* New York Heart Association, *NICM* non-ischaemic cardiomyopathy, *VO*_*2*_ oxygen consumption, *VE/VCO*_*2*_ CO_2_ exchange efficiency.

**Table 3:** Multivariable Cox regression concerning surrogate endpoints and MACE for the total population, non-ischaemic and ischaemic subpopulations

Potential surrogate endpoints	Multivariable HR (CI)		
	Total population	NICM	ICM
**∆ LVESV (%)**	0.975 (0.960–0.991)	0.960 (0.938–0.983)	–
**∆ LVEF (absolute %)**	–	–	–
**∆ VE/VCO**_**2**_ **slope**	–	–	–
**∆ BNP (pmol/L)**	–	–	0.993 (0.988–0.998)
**∆ NYHA (class)**	–	–	–

*∆* indicates a change, *BNP* brain natriuretic peptide, *ICM* ischaemic cardiomyopathy, *IVMD* interventricular mechanical delay, *LVEF* left ventricular ejection fraction, *LVESV* left ventricular end-systolic volume, *MACE* major adverse cardiac events, *NYHA* New York Heart Association, *NICM* non-ischaemic cardiomyopathy, *VO*_*2*_ oxygen consumption, *VE/VCO*_*2*_ CO_2_ exchange efficiency.

**Figure 3:**
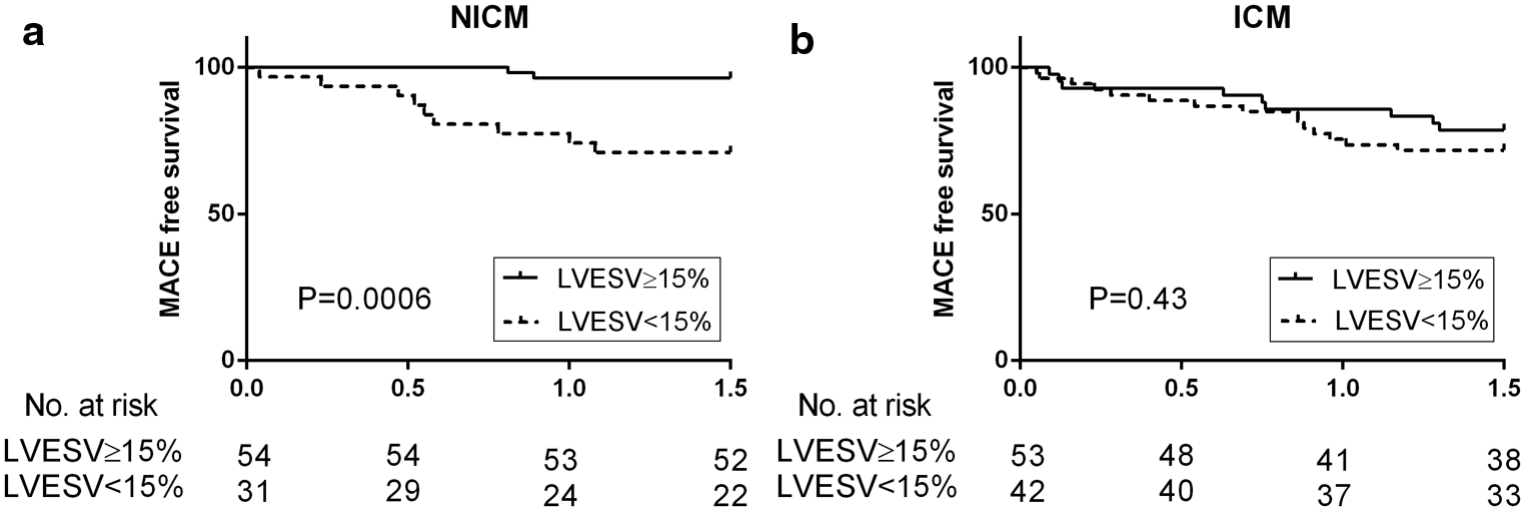
**a Kaplan-Meier estimates of survival in non-ischaemic subjects stratified by amount of left ventricular end-systolic volume decrease**. A 15 % cut-off for ∆LVESV was chosen, as this is the most often used cut-off value to discriminate between responders and non-responders [6, 30]. **b Kaplan-Meier estimates of survival in ischaemic subjects stratified by amount of left ventricular end systolic volume decrease**. A 15 % cut-off for ∆LVESV was chosen, as this is the most often used cut-off value to discriminate between responders and non-responders [6, 30]

Associations between surrogate response markers and MACE differed among heart failure aetiologies (Table 3, Table 4 and 5). In the non-ischaemic cardiomyopathy population results were in concordance with the total population as ∆LVESV remained the only surrogate marker for CRT response (Table 4). Discriminative power of ∆LVESV was high in this subpopulation: AUC = 0.85 (Fig. 1 and Fig. 3a).

**Table 4:** Comparison of changes in echocardiographic, cardiopulmonary, and laboratory parameters between patients with non-ischaemic cardiomyopathy with and without a MACE

Potential surrogate endpoints	MACE (*n* = 11)	Non-MACE (*n* = 74)	*P*-value
**∆ LVESV (%)**	− 2.4 ± 11.5	− 30.0 ± 24.2	< 0.001
**∆ LVEF (absolute %)**	3.5 ± 6.2	10.9 ± 9.3	0.013
**∆ IVMD (ms)**	− 35 ± 37	− 37 ± 34	0.832
**∆ QRS duration (ms)**	3.6 ± 28.0	17.6 ± 28.2	0.132
**∆ Peak VO**_**2**_ **(ml/kg/min)**	2.2 ± 2.9	1.36 ± 3.5	0.615
**∆ Predicted peak VO**_**2**_ **(absolute %)**	12.4 ± 15.1	6.7 ± 16.0	0.365
**∆ VE/VCO**_**2**_ **slope**	10 (19)	3 (6)	0.049
**∆ BNP (pmol/L)**	− 14 (360)	32 (106)	0.627
**∆ Creatinine (µmol/L)**	5.0 (29)	− 1.5 (22)	0.159
**NYHA improvement (%)**	6 (60)	50 (66)	0.718

*∆* indicates a change, *BNP* brain natriuretic peptide, *ICM* ischaemic cardiomyopathy, *IVMD* interventricular mechanical delay, *LVEF* left ventricular ejection fraction, *LVESV* left ventricular end-systolic volume, *MACE* major adverse cardiac events, *NYHA* New York Heart Association, *NICM* non-ischaemic cardiomyopathy, *VO*_*2*_ oxygen consumption, *VE/VCO*_*2*_ CO_2_ exchange efficiency.

**Table 5:** Comparison of changes in echocardiographic, cardiopulmonary, and laboratory parameters between patients with ischaemic cardiomyopathy with and without a MACE

Potential surrogate end-points	MACE (*n* = 24)	Non-MACE (*n* = 71)	*P*-value
**∆ LVESV (%)**	− 12.7 ± 20.5	− 16.8 ± 19.8	0.392
**∆ LVEF (absolute %)**	3.5 ± 6.3	5.5 ± 7.8	0.269
**∆ IVMD (ms)**	− 26 ± 35	− 31 ± 31	0.537
**∆ QRS duration (ms)**	4.3 ± 29.3	15.1 ± 24.3	0.101
**∆ Peak VO**_**2**_ **(ml/kg/min)**	0.1 ± 2.8	0.8 ± 2.5	0.299
**∆ Predicted peak VO**_**2**_ **(absolute %)**	2.7 ± 13.9	5.3 ± 13.6	0.496
**∆ VE/VCO**_**2**_ **slope**	0.7 (18)	4.4 (13)	0.139
**∆ BNP (pmol/L)**	35 (77)	− 53 (78)	0.041
**∆ Creatinine (µmol/L)**	− 6.0 (27)	− 5.0 (22)	0.673
**NYHA improvement (%)**	6 (27)	44 (60)	0.008

*∆* indicates a change, *BNP* brain natriuretic peptide, *ICM* ischaemic cardiomyopathy, *IVMD* interventricular mechanical delay, *LVEF* left ventricular ejection fraction, *LVESV* left ventricular end-systolic volume, *MACE* major adverse cardiac events, *NYHA* New York Heart Association, *NICM* non-ischaemic cardiomyopathy, *VO*_*2*_ oxygen consumption, *VE/VCO*_*2*_ CO_2_ exchange efficiency.

In the ischaemic cardiomyopathy population no significant correlation was found between ∆LVESV and the occurrence of MACE (Fig. 3b). In this group ∆BNP was the only independent surrogate marker for CRT response (Table 5). Discriminative power was moderate with AUC 0.66.

Concerning Fig. 2, a 15 % cut-off for ∆LVESV was chosen, as this is the most often used cut-off value to discriminate between responders and non-responders [6, 9].

## Discussion

For the total population, ∆LVESV was the most reliable surrogate marker for CRT response. Especially in the non-ischaemic cardiomyopathy population, this parameter showed excellent performance for discriminating between favourable and unfavourable long-term outcome. However, we demonstrated that ∆LVESV is a poor surrogate marker for CRT response in patients with ischaemic cardiomyopathy. In this group of patients ∆LVESV was unable to indicate which patients had a favourable and which had an unfavourable long-term outcome. These findings are of utmost importance as ∆LVESV is frequently chosen as outcome measure to assess response to CRT treatment regardless of aetiology [6, 13].

### Surrogate markers in non-ischaemic cardiomyopathy

For non-ischaemic cardiomyopathy, ∆LVESV was the best surrogate marker in our study. The importance of change in LV volume during CRT over other surrogate markers has been demonstrated previously and seems to be in concordance with our data [8, 14]. ∆LVESV was independent of change in LV systolic function, cardiopulmonary condition, mechanical dyssynchrony and cardiovascular biomarkers.

The clinical course of heart failure is determined by cardiac remodelling [15]. A decrease in LVESV implies reverse remodelling because it reflects both structural (reverse) remodelling and increased fibre shortening. LVEF, as a parameter for LV systolic function, seems less suitable as surrogate marker, probably because it is more dependent on LV end-diastolic volume and heart rate [15].

A change of mechanical dyssynchrony was not an appropriate surrogate outcome marker either. Mechanical dyssynchrony is not obligatory to be eligible for CRT implantation [16, 17]. Consequently, IVMD at baseline, and its reduction, showed a wide distribution in our study cohort, making ∆IVMD an unsuitable surrogate marker.

Cardiopulmonary exercise testing parameters may take longer to improve than 6 months. It has been demonstrated in heart failure patients that during a training programme time to reach maximum improvement varied between 16 and 26 weeks [18]. This process probably takes even longer without training, when it is solely influenced by CRT.

Also, neither ∆BNP nor ∆creatinine could identify those with a favourable or unfavourable long-term outcome in the non-ischaemic cardiomyopathy group. ∆BNP may not be reliable due to a wide distribution and high day-to-day variability [19]. Renal function does not seem to be affected by CRT as both groups showed only minor changes in creatinine between baseline and 6 months. This is in line with a recent study [20]. Moreover, when stratified according to baseline renal function, patients demonstrating either an improvement or decline in renal function occurred for all disease stages and changes were therefore unpredictable [20]. Consequently, changes in renal function can probably not discriminate between favourable and unfavourable clinical outcome.

### Surrogate markers in ischaemic cardiomyopathy

A remarkable finding was that ∆LVESV in ischaemic cardiomyopathy could not distinguish between patients who remained free of MACE during follow-up. Although ∆BNP was the best surrogate marker in this subpopulation, it was unsuitable for good discrimination. This implicates that ischaemic cardiomyopathy is influenced by other factors when it comes to long-term outcome.

Although both aetiologies are characterised by reduced contractility and global dilation, the disease substrate differs greatly. In ischaemic cardiomyopathy, coronary artery disease is the pathophysiological substrate and a high variety exists concerning the extent of disease, affecting prognosis, and making this a very heterogeneous group [21].

Furthermore, it has been demonstrated that scar tissue is a more important determinant of long-term outcome in ischaemic cardiomyopathy than contractile reserve [22]. This is also confirmed by the current study. As BNP is mainly produced by cardiomyocytes [23], it could be hypothesised that BNP change is only possible in patients with significant amounts of myocytes left, and consequently less scar tissue. Also, presence of myocardial scar in the pacing region was another important determinant of long-term outcome in patients with ischaemic cardiomyopathy eligible for CRT [24].

In addition, in about 25 % of patients disease extends beyond the coronary arteries and also affects the brain and peripheral tissues, which may affect prognosis independent of the amount of cardiac remodelling [25]. Therefore, it might be important to assess overall atherosclerotic status before CRT implantation. Importantly, these results do not imply that CRT is not beneficial for patients with ischaemic cardiomyopathy.

### Prediction of MACE

Although the present study searched for the best surrogate markers for CRT response through a correlation with MACE, prediction of MACE was not the aim. However, as the true endpoint of CRT is reduction of MACE, surrogate markers must be assessed on their capacity to discriminate between patients with high and low risk for future MACE [8, 9, 14]. In addition, a surrogate marker is a variable reflecting a change, for it should measure an effect of the delivered therapy [7].

When assessing risk of MACE independent of the effect of CRT, absolute values should also be taken into account, especially in the ischaemic cardiomyopathy group. In that case, besides ∆LVESV, also creatinine levels and peak VO_2_ at 6 months were demonstrated to be independent predictors of MACE. Both renal function and peak VO_2_ have prognostic value in heart failure patients [26–28].

Analysis was performed excluding appropriate ICD shocks as part of MACE. The discriminative value of ∆LVESV concerning the whole population, non-ischaemic and ischaemic cardiomyopathy subpopulations, did not significantly change (results not shown).

## Limitations

This study was a retrospective single-centre study with its inherent limitations. However, our cohort comprised a rather large number of patients, and MACE after 6 months occurred in 19 % of this cohort. MACE occurring before 6 months were not taken into account for this analysis, although these might show a relation with surrogate markers at 6 months. However, the first 6 months are a more instable phase as the effect of CRT might not yet be complete. In addition, the aim of the current study was to assess which surrogate marker at 6 months could best discriminate between patients who did and who did not encounter a MACE in the period thereafter. Moreover, LVESV and LVEF were assessed by echocardiograph. Recently De Haan et al. [29] demonstrated that eligibility to CRT depends on the imaging modality applied. This could also be true for assessment of response. This should be investigated in future research.

## Conclusion

∆LVESV is the most reliable surrogate marker for CRT response in the total population. This is attributed to the non-ischaemic cardiomyopathy subpopulation in which ∆LVESV showed an excellent correlation with long-term outcome. On the other hand, for ischaemic cardiomyopathy ∆LVESV showed a poor correlation with long-term outcome. Therefore cardiologists should be careful using volumetric response as a prognostic marker in ischaemic cardiomyopathy.

### Funding

None.
